# Multi-Port Robotic-Assisted Laparoscopic Myomectomy: A Systematic Review and Meta-Analysis of Comparative Clinical and Fertility Outcomes

**DOI:** 10.3390/jcm12124134

**Published:** 2023-06-19

**Authors:** Elias Tsakos, Emmanouil M. Xydias, Apostolos C. Ziogas, Felice Sorrentino, Luigi Nappi, Nikolaos Vlachos, Angelos Daniilidis

**Affiliations:** 1 EmbryoClinic IVF, 55133 Thessaloniki, Greece; tsakos@embryoclinic.eu (E.T.); manos.xydias@embryoclinic.eu (E.M.X.); 2 Faculty of Medicine, School of Health Sciences, University of Thessaly, 41500 Larissa, Greece; 3 Department of Medical and Surgical Sciences, Institute of Obstetrics and Gynaecology, University of Foggia, 71121 Foggia, Italy; luigi.nappi@unifg.it; 4 2nd Department of Obstetrics and Gynaecology, Aretaieio Hospital, School of Medicine, National and Kapodistrian University of Athens, 11528 Athens, Greece; 5 1st University Department of Obstetrics and Gynecology, Papageorgiou General Hospital, School of Medicine, Aristotle University of Thessaloniki, 54124 Thessaloniki, Greece; angedan@hotmail.com

**Keywords:** myomectomy, da Vinci, robotic gynaecologic surgery, uterine fibroids, minimally invasive surgery

## Abstract

Background: Uterine fibroids are the most frequently diagnosed gynaecological tumours, and they often require surgical treatment (conventional laparoscopic myomectomy—CLM). The introduction and evolution of robotic-assisted laparoscopic myomectomy (RALM) in the early 2000s has expanded the range of minimally invasive options for the majority of cases. This study aims to compare RALM with CLM and abdominal myomectomy (AM). Methods and materials: Fifty-three eligible studies adhered to the pre-established inclusion criteria and were subsequently evaluated for risk of bias and statistical heterogeneity. Results: The available comparative studies were compared using surgical outcomes, namely blood loss, complication rate, transfusion rate, operation duration, conversion to laparotomy, and length of hospitalisation. RALM was significantly superior to AM in all assessed parameters other than operation duration. RALM and CLM performed similarly in most parameters; however, RALM was associated with reduced intra-operative bleeding in patients with small fibroids and had lower rates of conversion to laparotomy, proving RALM as a safer overall approach. Conclusion: The robotic approach for surgical treatment of uterine fibroids is a safe, effective, and viable approach, which is constantly being improved and may soon acquire widespread adoption and prove to be superior to CLM in certain patient subgroups.

## 1. Introduction

Uterine fibroids, also referred to as leiomyomas or simply myomas, are amongst the most prevalent gynaecological disorders, with ultrasonographic findings indicative of their presence being detected in up to 80% of women by the age of 50 years [1]. While in the majority of cases they remain asymptomatic and are diagnosed incidentally [2], fibroids may present with pelvic pain, abnormal uterine bleeding, dysmenorrhea, and pressure effects, leading to disturbed urinary, gastrointestinal, and sexual function. More insidiously, they may also be the cause of secondary infertility, emotional distress, anxiety, or depression, with significant adverse effects on the overall quality of life [3]. Considering the above, effective treatment is paramount not only for symptom alleviation but also in order to improve future fertility prospects. While a plethora of non-invasive options for myoma management are available, surgical treatment remains the gold standard [4], with minimally invasive surgery in particular offering considerable advantages and currently being the most frequently preferred option [5]. Amongst the most advanced minimally invasive options is robotic-assisted laparoscopic myomectomy (RALM), offering impressive three-dimensional, magnified visualisation capabilities and a natural, finger-like, and intuitive control of the surgical instruments and superior ergonomics [6]. The aforementioned technical advantages may theoretically contribute to increased intra-operative efficiency, control, and safety and thus lead to fewer complications and morbidity and ultimately better patient outcomes. In this systematic review, we aim to test this hypothesis, namely whether these technical advantages improve RALM’s safety and effectiveness over conventional laparoscopic myomectomy (CLM) and abdominal myomectomy by laparotomy (AM).

## 2. Materials and Methods

This is a systematic review conducted based on pre-established criteria and on the methodology suggested by the Preferred Reporting Items for Systematic Reviews and Meta-Analyses (PRISMA 2020) Statement [7]. A formal study protocol was prepared a priori and was registered to the PROSPERO online database (CRD42023417412).

### 2.1. Eligibility Criteria

The research question at the core of this review was structured using the PICOS (population–intervention–comparator–outcome–study design) format in order to ensure that the data and outcomes searched for were precisely defined beforehand, which is a strategy also supported by the recommendations of the Cochrane Collaboration [8]. The target population of this review comprised women with uterine fibroids who underwent surgical treatment regardless of clinical manifestations. This study excluded paediatric populations and mesenchymal tumours arising at different anatomical regions. The intervention investigated was robotic-assisted laparoscopic myomectomy (RALM). Given the rise of various surgical systems and methodologies [9,10,11], the investigated intervention was further refined to the multi-port variant, defined as the use of at least one robotic port for camera placement and two additional robotic ports for the robotic arms and instruments, with the optional third robotic port and/or accessory port(s). Furthermore, the investigated robotic system was the da Vinci Surgical System by Intuitive, USA, due to its prevalence in the available literature and in order to facilitate the methodological homogeneity of the assessed studies. Comparators were not mandatory for study inclusion; however, they were crucial for comparative meta-analysis; thus, conventional laparoscopic myomectomy (CLM) and abdominal myomectomy (AM) were both included as eligible comparators. The primary sought outcomes of this study were the metrics of surgical performance consistent enough in the available literature to facilitate comparison of the approaches, namely mean operation duration (MOD), estimated blood loss (EBL), hospitalisation duration or length of stay (LOS), transfusion rate, complication rate, and conversion to laparotomy rate for RALM and CLM. The secondary outcomes included symptom improvement and fertility outcomes for women wishing to conceive. Acceptable study designs were either prospective or retrospective case series, cohorts, case-control studies, and clinical trials. Single-case reports or secondary studies were excluded from this analysis.

### 2.2. Search Methodology and Data Sources

Studies relevant to our research question were sought within the peer-reviewed medical research of *MEDLINE/PubMed*, *Web of Science*, and *Scopus*, with the most recent search having been conducted on 11/01/2023. The same search terms were used on all three databases: (robotic OR “robotic assisted” OR “robotic-assisted”) AND (myomectomy OR fibroidectomy). No search limitations or additional automated search filters were utilised during this process, with the results being manually refined by the investigators during the study assessment process.

### 2.3. Selection Methodology and Extracted Data

The resulting initial study pool was evaluated independently by two teams of authors who followed the same steps. Initially, duplicate records were removed, with the remaining records being screened for relevance and the most promising being moved to full-text assessment. Disagreements between the two independent teams were reviewed and resolved, with the included articles ultimately being agreed upon by all co-authors. Data on study design (study period, type of study, total sample size, and year of publication), patients’ characteristics (mean age, mean BMI, etc.), myoma characteristics (mean number, weight, diameter, etc.), RALM characteristics (da Vinci model, methodology, trocars used, etc.), and comparators were extracted and organised. This extraction process was performed by three authors who each analysed one-third of the studies, which were subsequently verified by another author for accuracy and consistency with the original article. 

With regard to sought outcomes, data on MOD, EBL, LOS, complication rates, transfusion rates, and conversion rates were extracted and utilised for meta-analysis. For the aforementioned meta-analysis, group sample sizes, means, and standard deviations were extracted and used. In order to include more studies and mitigate the effects of reporting bias, where enough data were provided, the means and standards deviations were estimated using the methods proposed by Luo et al. [12] and Shi et al. [13], whose models offer more accurate and robust estimation and are expanded and refined versions of previous similar works [14,15]. Additional data on fertility were also extracted and organised and are presented for the purposes of this review, although not in the form of meta-analysis. In the cases where the aforementioned outcomes were reported inconsistently, data were converted to the format used by the majority of the included studies or were excluded from our analysis if the former option was not feasible.

### 2.4. Risk of Bias Assessment

To facilitate the assessment of risk of bias amongst the included comparative studies, the Newcastle–Ottawa Scale (NOS) [16] for non-randomised studies was used. Two authors independently rated the studies using the aforementioned scale, and the results were compared for consistency. Any disagreements were resolved via re-evaluation or by the input of a third author.

### 2.5. Statistical Analysis

For the quantification and comparison of outcomes between RALM, CLM, and AM, specific effect measures were utilised, namely the weighted mean difference (WMD) for EBL, MOD, and LOS, while the odds ratio (OR) was used for transfusion rate, complication rate, and conversion to laparotomy rate. Data on the different outcomes were tabulated and compared between the intervention and the comparators.

The meta-analysis was conducted using the random effects model. Statistical heterogeneity between the studies was evaluated with the use of the I^2^ statistic, as formally suggested in the Cochrane Handbook for systematic reviews [8]. An I^2^ < 50% and a *p*-value over 0.05 were considered indicative of non-significant heterogeneity. The meta-analysis was conducted with the results either being pooled OR or WMD of the meta-analysed outcomes during comparisons.

A sensitivity analysis was conducted in order to assess the effect of risk of bias and of the inclusion of estimated results in the meta-analysis. Regarding the latter, while the applied predictive models offer remarkable accuracy for data following the normal distribution, the model’s performance remains suboptimal in cases of skewed data (non-normal distribution) [13]. Therefore, the effect of the inclusion of estimates from non-normally distributed data was assessed along with the effect of study risk of bias. Where possible and most relevant, forest plots were constructed to facilitate visualisation of the assessed correlations; otherwise, data are provided in text, tables, or the Supplementary Material. Calculations and graphs were carried out using the Stata Statistical Software: Release 14.2 by StataCorp LP.

## 3. Results

**Figure 1:**
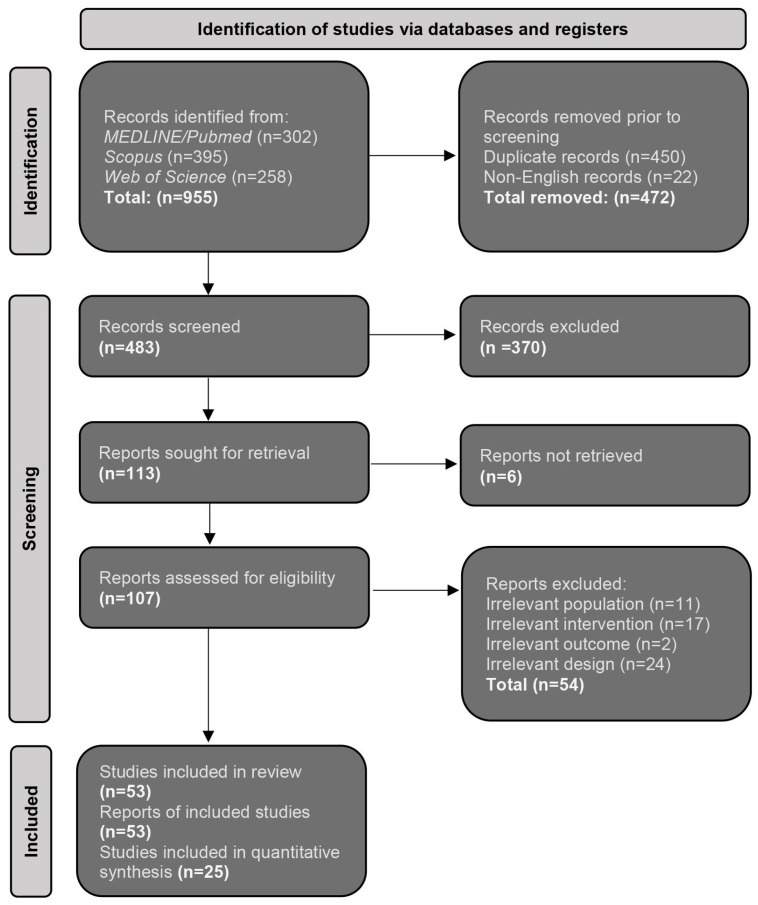
Summary flowchart of the evaluation process of the sought study, according to the PRISMA 2020 Guidelines.

The initial pool of 955 studies, which resulted from the preliminary search of the medical databases, was refined via manual screening of the title and abstract, leading to 107 reports being moved to full-text assessment. Through constant evaluation and strict application of the pre-established inclusion criteria, 53 studies were ultimately included in this review [17,18,19,20,21,22,23,24,25,26,27,28,29,30,31,32,33,34,35,36,37,38,39,40,41,42,43,44,45,46,47,48,49,50,51,52,53,54,55,56,57,58,59,60,61,62,63,64,65,66,67,68,69]. This systematic process of selection is summarised in Figure 1.

Most of the eligible studies originated from the USA, the Republic of Korea, and Taiwan, and the vast majority were based on data collected retrospectively from institutional medical records, with only eight studies being conducted prospectively. Twenty-five of the studies utilised an eligible comparator, i.e., CLM, AM, or both, and thus were utilized in comparative analysis. Ultimately, this systematic review included data from 7109 women. This information is summarised in Supplementary Table S1. The mean age of the included participants ranged from 34 to 48.2 years and mean BMI from 20.2 to 31 kg/m^2^. The main indications for myomectomy were clinical symptoms such as pressure effects associated with a pelvic mass, pelvic pain, uterine bleeding, infertility, gastrointestinal symptoms, urinary symptoms, etc., and a sizeable percentage of participants had a history of prior abdominal surgery and/or caesarean section (Supplementary Table S2). With regard to RALM technical characteristics, the majority of available studies used previous-generation da Vinci Surgical Systems, with only nine studies using the 4th generation da Vinci X and XI systems. With regard to the procedure, the basic steps were consistent amongst all studies. Most studies used a 12 mm robotic port for the camera, placed either above or below the umbilicus, with at least two additional and most commonly 8 mm robotic ports placed bilaterally from the abdominal midline. A few studies used a third 8 mm robotic port, and most researchers used an accessory 12, 10, or 5 mm port as well. The mean number of robotically excised myomas per study ranged from 1.5 to 7, as did the mean myoma diameter (3–8.3 cm), the mean diameter of the largest myoma (5–11 cm), and mean myoma weight (30–450 g) (Supplementary Table S3). With regard to the risk of bias assessment for the studies included in data synthesis, eight studies [17,20,30,35,50,53,59,62] received a rating of NOS 9, thirteen studies [22,23,24,25,28,29,31,36,37,39,60,64,68] received a rating of NOS 8, and four studies [18,49,51,54] received a rating of NOS 7. Overall, the results were indicative of the satisfactory methodological quality of the included studies and a low risk of bias for the majority of them.

A sensitivity analysis was conducted in order to assess the effect of the risk of bias assessment and the effect of including estimates from studies with non-normally distributed data. With regard to the former, some subgroups based on NOS score did demonstrate a loss of statistical significance of the overall effect; however, the trends remained consistent regardless of NOS score. The exception to that was a reversal of observed trends in LOS in the RALM versus CLM comparison, where studies with an NOS score of 9 showed that RALM offered shorter LOS compared to CLM, with this difference not being statistically significant in that there were no statistically significant differences in the examined parameters. Given the fact that only 1 out of 33 subgroups was affected by NOS score, its overall effect was considered negligible and as not affecting the robustness of the synthesised data. With regard to the effect of including estimates from studies with non-normally distributed data, there were no effects on the RALM versus AM comparison; however, on the RALM versus CLM comparison, both EBL and LOS were affected: the former with reversal of the observed trend (although non-statistically significant) and the latter with loss of significance. While no statistically significant changes in observed trends were noted, the fact that two out of six synthesised comparisons were affected in the RALM versus CLM comparison may indicate that inclusion of less reliable estimates affected the robustness of this synthesis. Therefore, for this comparison, these estimates were not included in the forest plots presented in this review. The results of all sensitivity analyses are available in Table 1.

**Figure 2:**
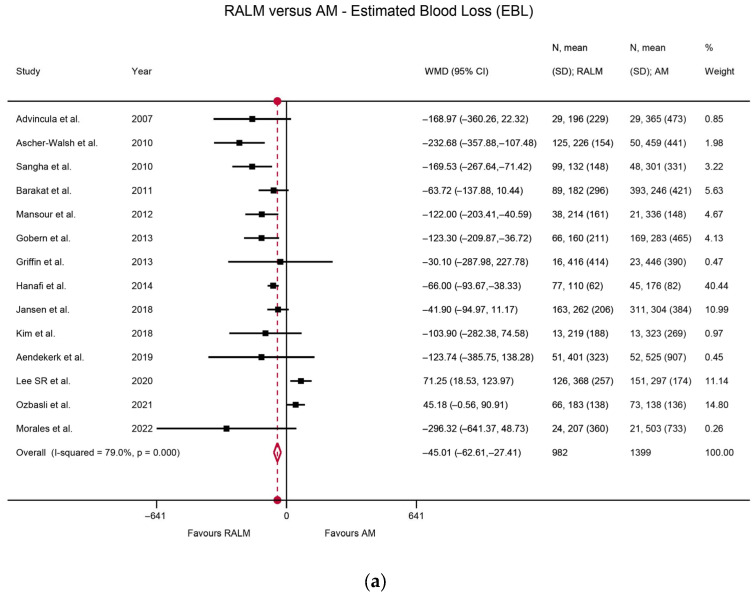

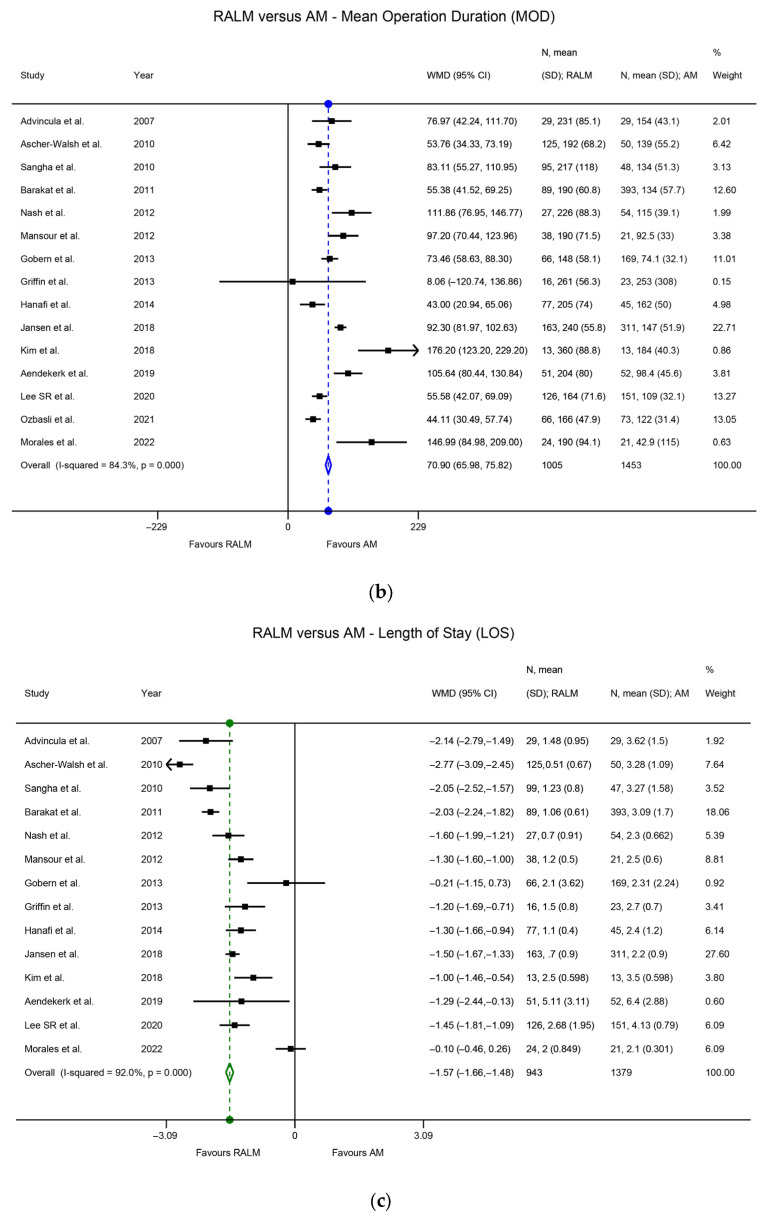
(**a**) Forest plot comparing RALM to AM on the basis of EBL [17,22,23,24,31,35,36,39,50,51,54,60,64,68]. (**b**) Forest plot comparing RALM to AM on the basis of MOD [17,22,23,24,25,31,35,36,39,50,51,54,60,64,68]. (**c**) Forest plot comparing RALM to AM on the basis of LOS [17,22,23,24,25,31,35,36,39,50,51,54,60,68].

**Figure 3:**
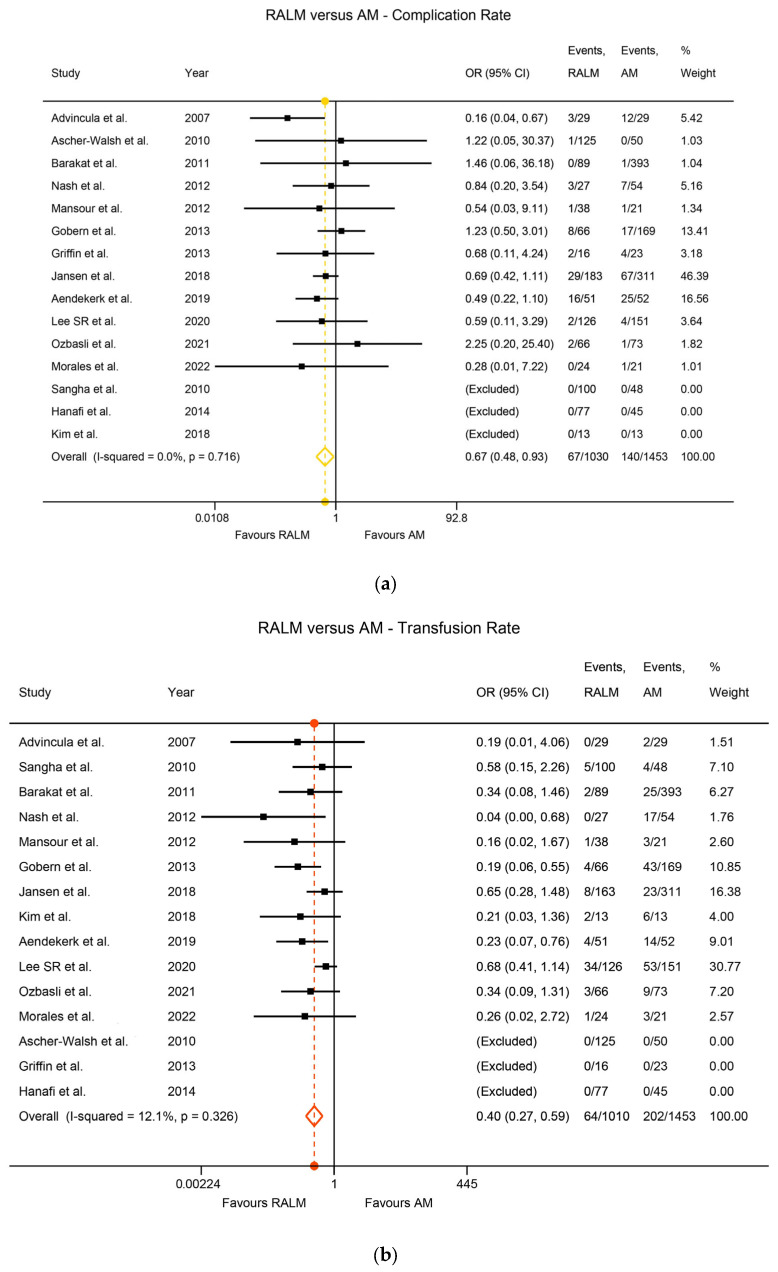
(**a**) Forest plot comparing RALM to AM on the basis of complication rate [17,22,23,24,25,31,35,36,39,50,51,54,60,64,68]. (**b**) Forest plot comparing RALM to AM on the basis of transfusion rate [17,22,23,24,25,31,35,36,39,50,51,54,60,64,68].

**Figure 4:**
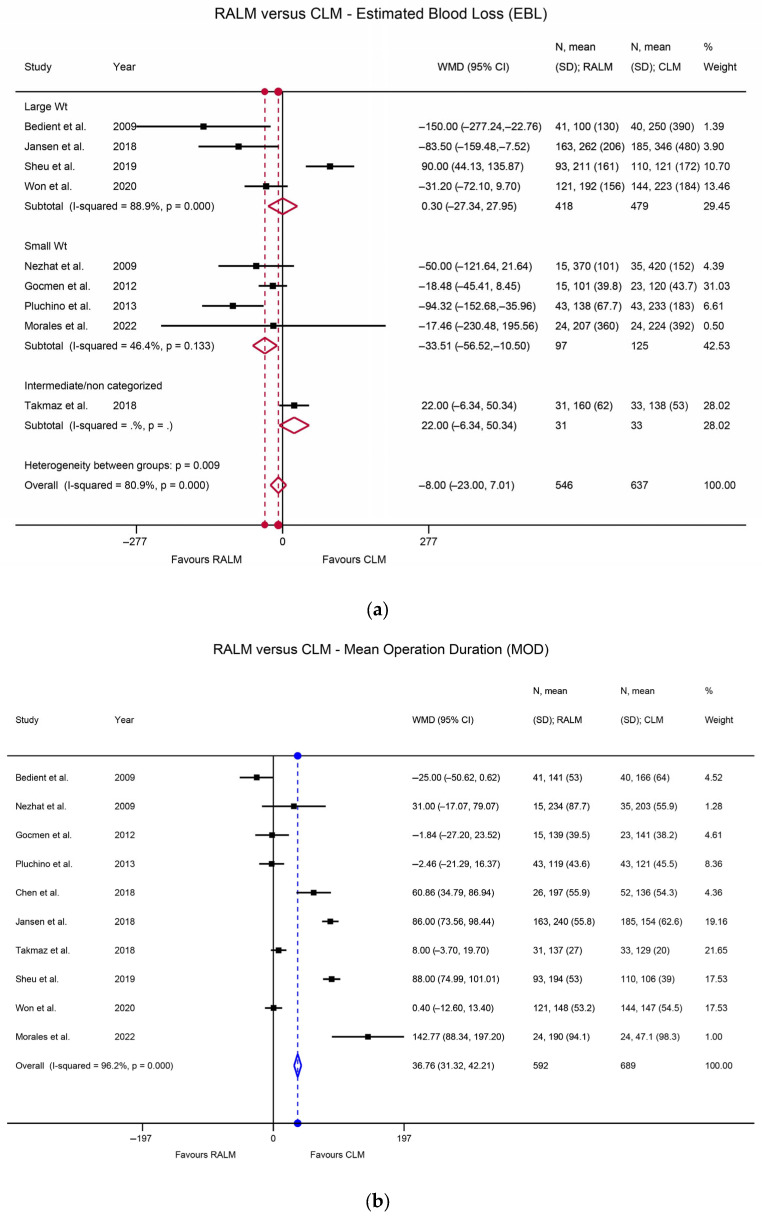

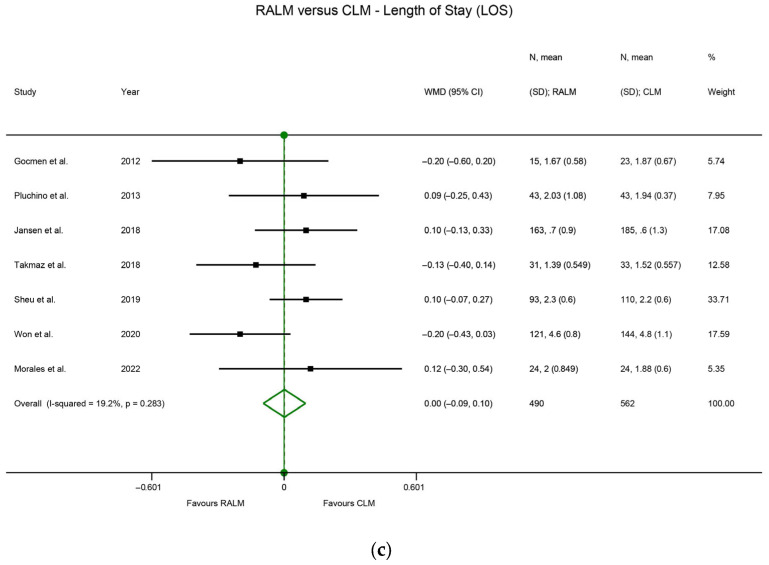
(**a**) Forest plot comparing RALM to CLM on the basis of EBL [18,20,29,37,50,53,59,62,68]. (**b**) Forest plot comparing RALM to CLM on the basis of MOD [18,20,29,37,49,50,53,59,62,68]. (**c**) Forest plot comparing RALM to CLM on the basis of LOS [29,37,50,53,59,62,68].

**Figure 5:**
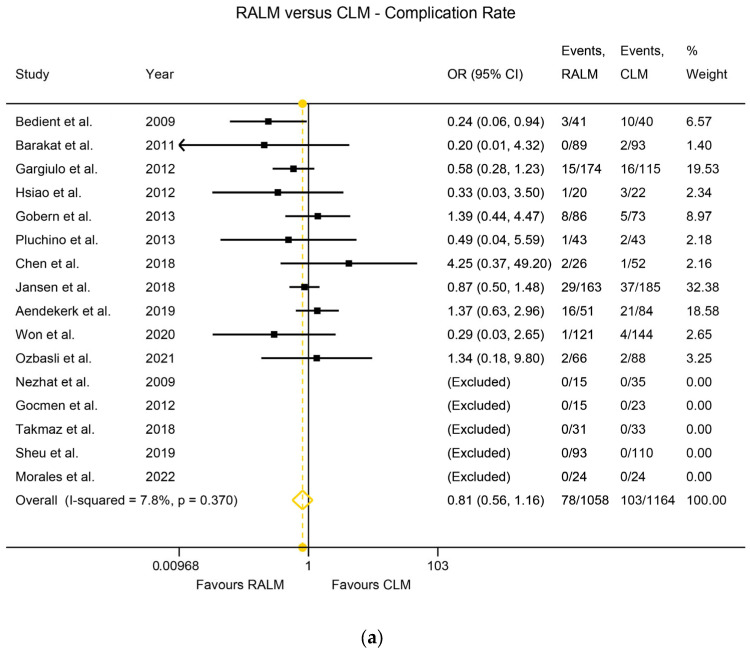

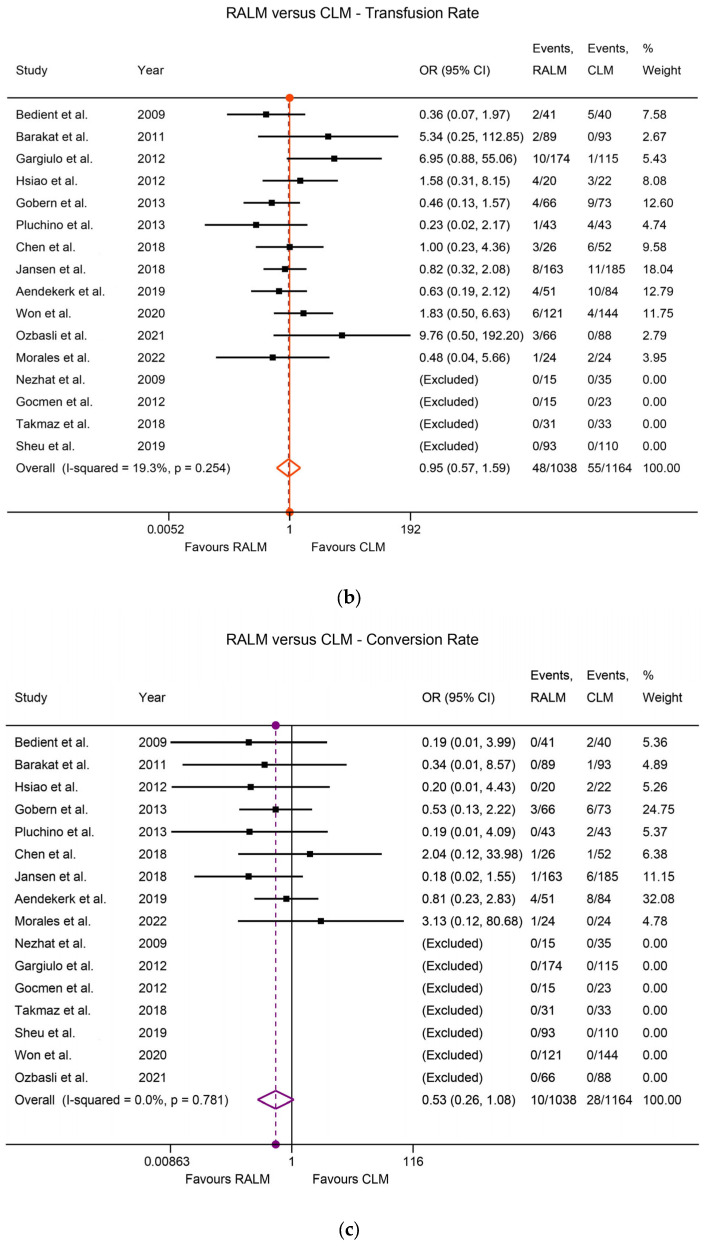
(**a**) Forest plot comparing RALM to CLM on the basis of complication rate [18,20,24,28,29,30,35,37,49,50,53,54,59,62,64,68]. (**b**) Forest plot comparing RALM to CLM on the basis of transfusion rate [18,20,24,28,29,30,35,37,49,50,53,54,59,62,64,68]. (**c**) Forest plot comparing RALM to CLM on the basis of conversion to laparotomy rate [18,20,24,28,29,30,35,37,49,50,53,54,59,62,64,68].

Heterogeneity among the studies was assessed using the I^2^ index. Regarding the RALM and AM comparison of primary surgical outcomes, there was statistically significant heterogeneity, namely I^2^ = 79.0% (*p* < 0.001) for the EBL comparison, I^2^ = 84.3% (*p* < 0.001) for the MOD comparison, and I^2^ = 92.0% (*p* < 0.001) for the LOS comparison. The heterogeneity was non-significant for the complication rate comparison (I^2^ = 0%, *p* = 0.716) and for the transfusion rate comparison (I^2^ = 12.1%, *p* = 0.326). With regard to actual outcomes, RALM had significantly lower EBL compared to AM, with WMD = 45.01 mL, *p* < 0.001 (Figure 2a). AM was superior with regard to MOD, requiring significantly shorter operation time, with WMD = 70.9 min, *p* < 0.001 (Figure 2b), and RALM necessitated a significantly shorter hospitalisation duration than AM, with the LOS WMD = 1.57 days, *p* < 0.001 (Figure 2c). RALM had a significantly lower complication rate compared to AM, being 33% safer, with OR = 0.67, *p* = 0.016 (Figure 3a), in addition to a 60% lower transfusion rate compared to AM, with a pooled OR = 0.4, *p* < 0.001 (Figure 3b). For the comparison of RALM to CLM, statistical heterogeneity was assessed for each comparison and was significant, with I^2^ = 80.9%, *p* < 0.001 for the EBL comparison and I^2^ = 96.2%, *p* < 0.001 for the MOD comparison. Heterogeneity was non-significant for the LOS comparison (I^2^ = 19.2%, *p* = 0.283), the complication rate comparison (I^2^ = 7.8%, *p* = 0.370), the transfusion rate comparison (I^2^ = 19.3%, *p* = 0.254), and the conversion to laparotomy rate comparison (I^2^ = 0%, *p* = 0.781). With regard to outcomes, RALM demonstrated lower EBL overall, but this was non-significant (WMD = 8 mL, *p* = 0.296); however, when the data were stratified according to myoma weight, RALM caused significantly less blood loss in cases with lower myoma weight (WMD = 33.51 mL, *p* = 0.004), while no statistically significant differences arose for the rest of the myoma cases (Figure 4a). This effect persisted in the analysis that included estimates from studies with skewed data distribution as well (WMD = 34.41, *p* = 0.003), while overall results differed. Similar to the RALM–AM comparison, RALM was inferior to CLM with regard to MOD, WMD = 36.76, *p* < 0.001 (Figure 4b), while there were no statistically significant differences between the two methods with regard to LOS, WMD = 0.002, *p* = 0.974 (Figure 4c). Complication and transfusion rates were similar in both methods, with no statistically significant differences, OR = 0.81, *p* = 0.250 and OR = 0.95, *p* = 0.854, respectively (Figure 5a,b). Conversion to laparotomy rate also technically showed no statistically significant difference between the two methods; however, the difference was only marginally non-significant, with OR = 0.53 and *p* = 0.083 (Figure 5c). With regard to fertility outcomes, pregnancy rates ranged from 50–80% postoperatively, with the majority of pregnancies originating from spontaneous conception. Studies with sufficient follow-up at the time of publication demonstrated a live birth rate of 25–100%.

Detailed fertility and obstetric outcomes are summarised in Table 2.

## 4. Discussion

In this systematic review, we examined the application of RALM, one of the newest minimally invasive available techniques, for the treatment of uterine fibroids and compared it to the other two established methodologies, namely AM and CLM. All available studies with data on RALM were collected and the information extracted and shown in tables. RALM compared favourably to AM in almost all aspects apart from operation duration.

Operation duration was also more favourable in CLM than RALM, with the rest of the assessed outcomes not being significantly different between CLM and RALM.

RALM was superior to CLM when estimated blood loss was assessed in lesser-myoma-burden patients. Regarding conversion to laparotomy rates, RALM was superior, although marginally not statistically significant. The findings of the present systematic review are indicative of the established trend of minimally invasive surgery adoption and expansion to further fields of gynaecologic surgery. Regarding the comparison between AM and RALM, our findings are in complete agreement with those of Wang et al. [70], who conducted a similar meta-analysis in 2018. However, we present different results when comparing RALM and CLM. 

**Figure 6:**
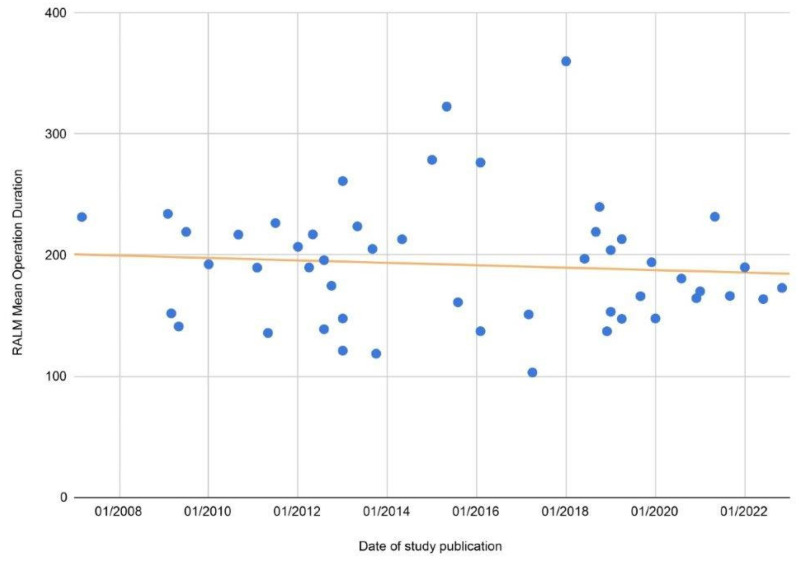
Scatter plot of the reported RALM MOD of all included studies, with a trend line depicting a downward trend as time passes.

In their analysis as well as in ours, Wang et al. showed that there were no statistically significant differences in transfusion rates or length of stay between RALM and CLM [70]. Additionally, they showed that there was a statistically significant difference in conversion to laparotomy rate [70] between the two methods. In our analysis, while conversion rate difference was non-significant, this was only so marginally; thus, the findings on conversion rates are actually quite similar. Wang et al. also showed statistically significant differences in complication rates, although when complications were analysed in subgroups, the differences were non-significant, which is similar to our general findings with regard to complications. Another difference between the two analyses was with regard to EBL, as Wang et al. showed there was a statistically significant difference between RALM and CLM regardless of other parameters. This was observed in our analysis only for lower-myoma-burden patients, with non-statistically significant overall difference. Finally, with regard to operative time, Wang et al. showed that there were no statistically significant differences, while in our analysis, CLM was significantly faster. However, regarding this particular comparison, in the meta-analysis by Wang et al., the difference was only marginally statistically significant [70]. These differences between the two studies may be attributed to differences in baseline characteristics of the patients, as in multiple studies, the two groups had statistically significant difference with regard to myoma number, size, weight, etc. Additionally, since the more modern studies also included patients treated with the 4th generation da Vinci Surgical Systems, the observed discrepancies may be attributed to the learning curve of the healthcare teams during these first reported cases. Furthermore, in both analyses, there was statistically significant heterogeneity amongst the included primary comparisons, and thus, the results of the meta-analysis might be affected in both cases. This heterogeneity may arise due to many studies reporting on their initial experience with RALM, thus lacking a period of familiarisation with the robotic system, with outcomes likely to improve as surgeons gain experience and higher-quality training. In our study, the unfavourable comparison of RALM with CLM and AM regarding operation duration, while indeed constituting a notable drawback, may be acceptable if morbidity risk is significantly reduced. Additionally, based on the data collected during the present study, RALM operation duration is following a downward trend already (Figure 6) and is likely to continue to do so in the future given the continuous evolution and applicability of robotic technology [70], improvement of training, and healthcare team experience. Compared to CLM, RALM proved to be superior with regard to estimated blood loss in patients with smaller overall myoma weight.

These cases usually include patients with smaller myomas, often deep into the myometrium, a situation where the absolute control and precision that the robotic surgical system offers is best-utilised, minimising surgical trauma and thus resulting in reduced blood loss. On the other hand, CLM was superior in cases with higher-weight myomas in terms of EBL (a difference that was statistically significant in the models that included all studies regardless of quality or data skewness). This was also confirmed by the fact that mean weight and size of removed fibroids were larger in the CLM groups compared to RALM groups in the primary studies, indicating that the researchers preferred CLM for very large fibroids. This may be attributed to increased surgeon experience with CLM, better training, or choice of patients with more superficially located myomas. Since myoma location is not consistently reported, the latter assumption remains unverified. Additionally, the fact that RALM offered up to 50% reduced risk of conversion to laparotomy, a complication associated with further, severe complications and an overall more adverse outcome [71,72], reinforces the safety and risk-minimisation aspect emphasised by robotic technology.

Robotic surgery has been a rapidly developing field in the 21st century, particularly with regards with gynaecologic surgery. The robotic equipment has been designed to surpass the limitation of conventional laparoscopy, providing superior, three-dimensional visualisation of the surgical site; increased magnification; and enhanced dexterity via highly articulating surgical instruments and absent tremor [73,74]. Additionally, the ergonomic working configuration of the surgical console ensures reduced strain, be it physical or mental, for the surgeon, with significant improvement to surgery ergonomics [75,76] and thus maintaining high levels of surgical performance, a vital feature especially for high-volume surgeons and/or multi-hour procedures. Given the prevalence of uterine fibroids and the effect that they may have on quality of life and fertility, a safe, effective, reliable, ergonomic, and consistent surgical approach such as RALM is a necessary and beneficial addition to the modern gynaecologic surgeon’s options [77]. Research data on the applicability of robotic systems in gynaecologic surgery and in fibroid management in particular are still lacking. Future research efforts should aim to design a randomised trial for RALM and CLM comparison, as such a study has not been conducted yet and would provide valuable scientific evidence. Furthermore, laparoscopically assisted myomectomy by mini-laparotomy is a popular alternative technique for minimally invasive myomectomy, which, despite being beyond the scope of this review, warrants further exploration and analysis, as it may be a beneficial option for certain patient subgroups with very large-volume fibroids. Additionally, further specialised applications and indications for robotic surgery should be sought after, as such data may be utilised in updating management algorithms and promoting robotic surgery to patients who truly stand to gain the most from this advanced method. The present study does come with certain limitations, which should be acknowledged. Firstly, there was statistically significant heterogeneity in the pooled available data, which may have introduced bias, affecting the results of the analysis. Additionally, there were not enough studies utilising the latest advances in robotic technology, such as the 4th generation da Vinci Surgical System, which may be more representative of the capabilities of modern technology since robotic surgery is such a rapidly evolving field. Thirdly, the two separate analyses conducted to assess the robustness of the synthesised data demonstrated deviations from each other with regard to EBL and LOS in the RALM versus CLM comparison; however, the results of both analyses were reported in order to reduce bias and ensure methodological transparency. Finally, not enough primary data were available to further stratify patients and thus to test the performance of the three therapeutic approaches in more specific cases.

## 5. Conclusions

In this systematic review and meta-analysis, we demonstrated that RALM was a superior option to AM in every assessed parameter apart from MOD. RALM was equal to CLM with regard to overall EBL, LOS, transfusion rate, and complication rate, with RALM being superior in EBL as observed in smaller-myoma-burden patients and conversion to laparotomy rate, with the latter, however, being marginally non-significant. CLM was superior with regard to MOD and was the preferred option in larger uterine fibroids. Despite their current limitations, robotic surgery technology as well as surgical skill are likely to further improve in the future, thus enhancing the benefits already offered by this approach. Future research should further examine the performance of robotic surgery in comparison with other minimally invasive options for myomectomy, in particular with laparoscopically assisted mini-laparotomy, for cases with very large myomas. Furthermore, researchers should focus on establishing specific patient subgroups that would most benefit from the robotic surgical approach, thus allowing for its more effective application in clinical practice.

## Figures and Tables

**Table 1:** Summary of results from the sensitivity analysis for robustness of data synthesis (CmpR, complication rate; TR, transfusion rate; CnvR, conversion to laparotomy rate; NOS, Newcastle–Ottawa scale).

	RALM vs. AM					RALM vs. CLM					
Parameter Assessed	EBL WMD (mL), *p*-Value	MOD WMD (min), *p*-Value	LOS WMD (Days), *p*-Value	CmpR OR, *p*-Value	TR OR, *p*-Value	EBL WMD (mL), *p*-Value	MOD WMD (min), *p*-Value	LOS WMD (Days), *p*-Value	CmpR OR, *p*-Value	TR OR, *p*-Value	CnvR OR, *p*-Value)
All studies	−45.012, *p* < 0.001 I^2^ = 79.0%, *p* < 0.001	70.898 *p* < 0.001 I^2^ = 84.3%, *p* < 0.001	−1.569 *p* < 0.001 I^2^ = 92.0%, *p* < 0.001	0.669 *p* = 0.016 I^2^ = 0.0%, *p* = 0.716	0.402 *p* < 0.001 I^2^ = 12.1%, *p* = 0.326	22.847 *p* < 0.001 I^2^ = 79.7%, *p* < 0.001	61.339 *p* < 0.001 I^2^ = 96.3%, *p* < 0.001	0.083 *p* = 0.018 I^2^ = 84.5%, *p* < 0.001	0.808 *p* = 0.250 I^2^ = 7.8%, *p* = 0.370	0.953 *p* = 0.854 I^2^ = 19.3%, *p* = 0.254	0.533 *p* = 0.083 I^2^ = 0.0%, *p* = 0.083
NOS 9 studies	−69.688 *p* = 0.002 I^2^ = 43.8%, *p* = 0.169	85.635 *p* < 0.001 I^2^ = 54.8%, *p* = 0.109	−1.502 *p* < 0.001 I^2^ = 81.9%, *p* = 0.004	0.612 *p* = 0.256 I^2^ = 64.8%, *p* = 0.059	0.351 *p* = 0.035 I^2^ = 42.7%, *p* = 0.175	7.953 *p* = 0.411 I^2^ = 80.9%, *p* < 0.001	47.334 *p* < 0.001 I^2^ = 97.2%, *p* < 0.001	−0.037 *p* = 0.397 I^2^ = 81.7%, *p* < 0.001	0.857 *p* = 0.519 I^2^ = 0.0%, *p* = 0.528	0.926 *p* = 0.801 I^2^ = 0.0%, *p* = 0.423	0.352 *p* = 0.065 I^2^ = 0.0%, *p* = 0.065
NOS 8 studies	−39.118 *p* < 0.001 I^2^ = 85.7%, *p* < 0.001	58.314 *p* < 0.001 I^2^ = 74.4%, *p* < 0.001	−1.637 *p* < 0.001 I^2^ = 94.5%, *p* < 0.001	0.799 *p* = 0.566 I^2^ = 0.0%, *p* = 0.981	0.472 *p* = 0.003 I^2^ = 9.6%, *p* = 0.356	24.059 *p* < 0.001 I^2^ = 83.4%, *p* < 0.001	68.855 *p* < 0.001 I^2^ = 95.9%, *p* < 0.001	0.165 *p* = 0.023 I^2^ = 36.8%, *p* = 0.191	0.601 *p* = 0.129 I^2^ = 0.0%, *p* = 0.766	1.891 *p* = 0.425 I^2^ = 48.5%, *p* = 0.101	0.562 *p* = 0.538 I^2^ = 0.0%, *p* = 0.538
NOS 7 studies	−110.187 *p* = 0.143 I^2^ = 0.0%, *p* = 0.902	118.651 *p* < 0.001 I^2^ = 82.0%, *p* = 0.018	−1.039 *p* < 0.001 I^2^ = 0.0%, *p* = 0.653	0.494 *p* = 0.085 I^2^ = NA	0.225 *p* = 0.004 I^2^ = 0.0%, *p* = 0.940	12.614 *p* = 0.707 I^2^ = 80.1%, *p* < 0.001	36.722 *p* < 0.001 I^2^ = 94.1%, *p* < 0.001	0.594 *p* < 0.001 I^2^ = 83.9%, *p* = 0.013	0.939 *p* = 0.931 I^2^ = 67.9%, *p* = 0.044	0.638 *p* = 0.283 I^2^ = 0.0%, *p* = 0.671	0.773 *p* = 0.638 I^2^ = 0.0%, *p* = 0.638
Studies with outcome estimates from skewed data removed	−50.848 *p* < 0.001 I^2^ = 80.7%, *p* < 0.001	70.848 *p* < 0.001 I^2^ = 86.9%, *p* < 0.001	−1.458 *p* < 0.001 I^2^ = 93.0%, *p* < 0.001	0.669 *p* = 0.016 I^2^ = 0.0%, *p* = 0.716	0.402 *p* < 0.001 I^2^ = 12.1%, *p* = 0.326	−7.995 *p* = 0.296 I^2^ = 80.9%, *p* < 0.001	36.762 *p* < 0.001 I^2^ = 96.2%, *p* < 0.001	0.002 *p* = 0.974 I^2^ = 19.2%, *p* = 0.283	0.808 *p* = 0.250 I^2^ = 7.8%, *p* = 0.370	0.953 *p* = 0.854 I^2^ = 19.3%, *p* = 0.254	0.533 *p* = 0.083 I^2^ = 0.0%, *p* = 0.781

**Table 2:** Fertility and obstetrics outcomes reported by included studies (CPR, clinical pregnancy rate; LBR, live birth rate; CS, caesarean section; NR, not reported).

Study	Year	Duration	Patients Aiming to Conceive	Conception		CPR	Time to Conception (Months)	Pregnancy Comp/Pathology	Miscarriage	Delivery Timing		Delivery Mode		LBR	Delivery Complications
				Spontaneous	ART					Full Term	Pre-Term	Vaginal	CS		
Lonnerfors et al. [26]	2011	04/2006–07/2010	22	18	3	68.2%	10	0	5	10	0	5	5	67%	0
Cela et al. [27]	2012	06/2007–03/2011	9	7	0	78%	16	0	0	7	0	2	5	100%	0
Pitter et al. [32]	2012	10/2005–11/2010	NR	77	50	NR	NR	17	24	0	16	2	88	NR	13
Tusheva et al. [34]	2012	01/2006–05/2009	16	12	4	50%	1–6	0	1	9	2	4	11	93.8%	2
Asmar et al. [40]	2015	01/2011–10/2014	5	2	2	80%	6	0	1	3	0	0	3	25%	0
Pitter et al. [43]	2015	08/2005–11/2013	63	17	15	50.8%	8	0	12	NR	NR	NR	NR	NR	NR
Kang et al. [45]	2016	04/2009–10/2013	12	9	0	75%	NR	0	1	7	0	0	7	100%	0
Flyckt et al. [46]	2016	01/1995–12/2009	15	5	3	53.3%	NR	0	0	NR	NR	0	5	100%	3
Huberland et al. [56]	2019	07/2009–04/2016	49	20	8	57.1%	17	0	4	21	1	7	17	85.7%	2
Park SU et al. [61]	2020	07/2015–03/2018	15	10	2	80%	NR	0	1	10	0	0	10	83.3%	1
Goldberg et al. [66]	2021	10/2008–09/2015	63	22	23	71.4%	NR	10	1	33	5	0	29	64.4%	1
Morales et al. [68]	2022	2010–2018	24	5	NR	58.3%	48	NR	1	NR	NR	4	0	80%	0

## Data Availability

The data presented in this study are available in this article and its Supplementary Materials.

## Supplementary Materials

The following supporting information can be downloaded at: https://www.mdpi.com/article/10.3390/jcm12124134/s1, Table S1: Baseline characteristics of the included studies (N/A, not applicable; RALM, robotic-assisted laparoscopic myomectomy; CLM, conventional laparoscopic myomectomy; AM, abdominal myomectomy); Table S2: Baseline and clinical patient characteristics. (NR, not reported; * the same study divided in two because of different groups; RALM, robotic-assisted laparoscopic myomectomy; CLM, conventional laparoscopic myomectomy; AM, abdominal myomectomy); Table S3: Da Vinci Surgical System and procedure characteristics and fibroid baseline characteristics (MMN, mean myoma number; MMD, mean myoma diameter; MDLM, mean diameter of largest myomas; MMW, mean myoma weight; NR, not reported). References [17,18,19,20,21,22,23,24,25,26,27,28,29,30,31,32,33,34,35,36,37,38,39,40,41,42,43,44,45,46,47,48,49,50,51,52,53,54,55,56,57,58,59,60,61,62,63,64,65,66,67,68,69] are cited in the Supplementary Materials.

## References

[1] Baird D., Dunson D.B., Hill M.C., Cousins D., Schectman J.M. (2003). High cumulative incidence of uterine leiomyoma in black and white women: Ultrasound evidence. Am. J. Obstet. Gynecol..

[2] Giuliani E., As-Sanie S., Marsh E.E. (2020). Epidemiology and management of uterine fibroids. Int. J. Gynecol. Obstet..

[3] Ghant M.S., Sengoba K.S., Recht H., Cameron K.A., Lawson A.K., Marsh E.E. (2015). Beyond the physical: A qualitative assessment of the burden of symptomatic uterine fibroids on women’s emotional and psychosocial health. J. Psychosom. Res..

[4] Sabry M., Al-Hendy A. (2012). Medical Treatment of Uterine Leiomyoma. Reprod. Sci..

[5] Flyckt R., Coyne K., Falcone T. (2017). Minimally Invasive Myomectomy. Clin. Obstet. Gynecol..

[6] Moon A.S., Garofalo J., Koirala P., Vu M.-L.T., Chuang L. (2020). Robotic Surgery in Gynecology. Surg. Clin. N. Am..

[7] Page M.J., McKenzie J.E., Bossuyt P.M., Boutron I., Hoffmann T.C., Mulrow C.D., Shamseer L., Tetzlaff J.M., Akl E.A., Brennan S.E. (2021). The PRISMA 2020 Statement: An Updated Guideline for Reporting Systematic Reviews. BMJ.

[8] Reitsma J.B., Rutjes A.W.S., Whiting P., Vlassov V.V., Leeflang M.M.G., Deeks J.J. (2009). Chapter 9: Assessing methodological quality. Cochrane Handbook for Systematic Reviews of Diagnostic Test Accuracy.

[9] Alip S.L., Kim J., Rha K.H., Han W.K. (2022). Future Platforms of Robotic Surgery. Urol. Clin. N. Am..

[10] Dobbs R.W., Halgrimson W.R., Talamini S., Vigneswaran H.T., Wilson J.O., Crivellaro S. (2019). Single-port robotic surgery: The next generation of minimally invasive urology. World J. Urol..

[11] Guo N., Liu H. (2022). Robotic laparoendoscopic single-site gynecologic surgery. Asian J. Surg..

[12] Luo D., Wan X., Liu J., Tong T. (2018). Optimally estimating the sample mean from the sample size, median, mid-range, and/or mid-quartile range. Stat. Methods Med. Res..

[13] Shi J., Luo D., Weng H., Zeng X., Lin L., Chu H., Tong T. (2020). Optimally estimating the sample standard deviation from the five-number summary. Res. Synth. Methods.

[14] Wan X., Wang W., Liu J., Tong T. (2014). Estimating the sample mean and standard deviation from the sample size, median, range and/or interquartile range. BMC Med. Res. Methodol..

[15] Hozo S.P., Djulbegovic B., Hozo I. (2005). Estimating the mean and variance from the median, range, and the size of a sample. BMC Med. Res. Methodol..

[16] Stang A. (2010). Critical evaluation of the Newcastle-Ottawa scale for the assessment of the quality of nonrandomized studies in meta-analyses. Eur. J. Epidemiol..

[17] Advincula A.P., Xu X., Goudeau S., Ransom S.B. (2007). Robot-assisted laparoscopic myomectomy versus abdominal myomectomy: A comparison of short-term surgical outcomes and immediate costs. J. Minim. Invasive Gynecol..

[18] Bedient C.E., Magrina J.F., Noble B.N., Kho R.M. (2009). Comparison of robotic and laparoscopic myomectomy. Am. J. Obstet. Gynecol..

[19] George A., Eisenstein D., Wegienka G. (2009). Analysis of the Impact of Body Mass Index on the Surgical Outcomes after Robot-Assisted Laparoscopic Myomectomy. J. Minim. Invasive Gynecol..

[20] Nezhat C., Lavie O., Hsu S., Watson J., Barnett O., Lemyre M. (2009). Robotic-assisted laparoscopic myomectomy compared with standard laparoscopic myomectomy—A retrospective matched control study. Fertil. Steril..

[21] Piquion-Joseph J.M., Nayar A., Ghazaryan A., Papanna R., Klimek W., Laroia R. (2009). Robot-assisted gynecological surgery in a community setting. J. Robot. Surg..

[22] Ascher-Walsh C.J., Capes T.L. (2010). Robot-assisted Laparoscopic Myomectomy Is an Improvement Over Laparotomy in Women with a Limited Number of Myomas. J. Minim. Invasive Gynecol..

[23] Sangha R., Eisenstein D.I., George A., Munkarah A., Wegienka G. (2010). Surgical outcomes for robotic-assisted laparoscopic myomectomy compared to abdominal myomectomy. J. Robot. Surg..

[24] Barakat E.E., Bedaiwy M.A., Zimberg S., Nutter B., Nosseir M., Falcone T. (2011). Robotic-Assisted, Laparoscopic, and Abdominal Myomectomy: A Comparison of Surgical Outcomes. Obstet. Gynecol..

[25] Nash K., Feinglass J., Zei C., Lu G., Mengesha B., Lewicky-Gaupp C., Lin A. (2012). Robotic-assisted laparoscopic myomectomy versus abdominal myomectomy: A comparative analysis of surgical outcomes and costs. Arch. Gynecol. Obstet..

[26] Lönnerfors C., Persson J. (2011). Pregnancy following robot-assisted laparoscopic myomectomy in women with deep intramural myomas. Acta Obstet. Gynecol. Scand..

[27] Cela V., Freschi L., Simi G., Tana R., Russo N., Artini P.G., Pluchino N. (2013). Fertility and endocrine outcome after robot-assisted laparoscopic myomectomy (RALM). Gynecol. Endocrinol..

[28] Gargiulo A.R., Srouji S.S., Missmer S.A., Correia K., Vellinga T.T., Einarsson J.I. (2012). Robot-Assisted Laparoscopic Myomectomy Compared with Standard Laparoscopic Myomectomy. Obstet. Gynecol..

[29] Göçmen A., Şanlıkan F., Uçar M.G. (2013). Comparison of robotic-assisted laparoscopic myomectomy outcomes with laparoscopic myomectomy. Arch. Gynecol. Obstet..

[30] Hsiao S.-M., Lin H.-H., Peng F.-S., Jen P.-J., Hsiao C.-F., Tu F.-C. (2013). Comparison of robot-assisted laparoscopic myomectomy and traditional laparoscopic myomectomy. J. Obstet. Gynaecol. Res..

[31] Mansour F.W., Kives S., Urbach D.R., Lefebvre G. (2012). Robotically Assisted Laparoscopic Myomectomy: A Canadian Experience. J. Obstet. Gynaecol. Can..

[32] Pitter M.C., Gargiulo A.R., Bonaventura L.M., Lehman J.S., Srouji S.S. (2013). Pregnancy outcomes following robot-assisted myomectomy. Hum. Reprod..

[33] Tan S.-J., Lin C.-K., Fu P.-T., Liu Y.-L., Sun C.-C., Chang C.-C., Yu M.-H., Lai H.-C. (2012). Robotic surgery in complicated gynecologic diseases: Experience of Tri-Service General Hospital in Taiwan. Taiwan. J. Obstet. Gynecol..

[34] Tusheva O.A., Gyang A., Patel S.D. (2013). Reproductive outcomes following robotic-assisted laparoscopic myomectomy (RALM). J. Robot. Surg..

[35] Gobern J.M., Rosemeyer C.J., Barter J.F., Steren A.J. (2013). Comparison of Robotic, Laparoscopic, and Abdominal Myomectomy in a Community Hospital. JSLS.

[36] Griffin L., Feinglass J., Garrett A., Henson A., Cohen L., Chaudhari A., Lin A. (2013). Postoperative Outcomes after Robotic Versus Abdominal Myomectomy. JSLS.

[37] Pluchino N., Litta P., Freschi L., Russo M., Simi G., Santoro A.N., Angioni S., Gadducci A., Cela V. (2014). Comparison of the initial surgical experience with robotic and laparoscopic myomectomy. Int. J. Med. Robot. Comput. Assist. Surg..

[38] Goetgheluck J., Carbonnel M., Ayoubi J.M. (2014). Robotically Assisted Gynecologic Surgery: 2-Year Experience in the French Foch Hospital. Front. Surg..

[39] Hanafi M. (2014). Comparative study between robotic laparoscopic myomectomy and abdominal myomectomy. Middle East Fertil. Soc. J..

[40] Asmar J., Even M., Carbonnel M., Goetgheluck J., Revaux A., Ayoubi J.M. (2015). Myomectomy by Robotically Assisted Laparoscopic Surgery: Results at Foch Hospital, Paris. Front. Surg..

[41] Cheng H.-Y., Chen Y.-J., Wang P.-H., Tsai H.-W., Chang Y.-H., Twu N.-F., Juang C.-M., Wu H., Yen M.-S., Chao K.-C. (2015). Robotic-assisted laparoscopic complex myomectomy: A single medical center’s experience. Taiwan. J. Obstet. Gynecol..

[42] Yim G.W., Kim S.W., Nam E.J., Kim S., Kim Y.T. (2015). Perioperative Complications of Robot-Assisted Laparoscopic Surgery Using Three Robotic Arms at a Single Institution. Yonsei Med. J..

[43] Pitter M.C., Srouji S.S., Gargiulo A.R., Kardos L., Seshadri-Kreaden U., Hubert H.B., Weitzman G.A. (2015). Fertility and Symptom Relief following Robot-Assisted Laparoscopic Myomectomy. Obstet. Gynecol. Int..

[44] Gunnala V., Setton R., Pereira N., Huang J.Q. (2016). Robot-Assisted Myomectomy for Large Uterine Myomas: A Single Center Experience. Minim. Invasive Surg..

[45] Kang S.Y., Jeung I.-C., Chung Y.-J., Kim H.-K., Lee C.R., Mansukhani T.S., Kim M.-R. (2017). Robot-assisted laparoscopic myomectomy for deep intramural myomas. Int. J. Med. Robot. Comput. Assist. Surg..

[46] Flyckt R., Soto E., Nutter B., Falcone T. (2016). Comparison of Long-Term Fertility and Bleeding Outcomes after Robotic-Assisted, Laparoscopic, and Abdominal Myomectomy. Obstet. Gynecol. Int..

[47] Liu W.-M., Chen C.-H., Chen H.-H. (2017). Complication reports for robotic surgery using three arms by a single surgeon at a single institution. J. Minimal Access Surg..

[48] Nam S.H., Paek J., Choi C., Nam S.H., Kim W.Y. (2017). A comparison between reduced-port robotic surgery and multiport robot-assisted laparoscopy for myomectomy. Eur. J. Obstet. Gynecol. Reprod. Biol..

[49] Chen Y.-C., Lin H.-H., Hsiao S.-M. (2018). Comparison of robotic assisted laparoscopic myomectomy with barbed sutures and traditional laparoscopic myomectomy with barbed sutures. Taiwan. J. Obstet. Gynecol..

[50] Jansen L.J., Clark N.V., Dmello M., Gu X., Einarsson J.I., Cohen S.L. (2019). Perioperative Outcomes of Myomectomy for Extreme Myoma Burden: Comparison of Surgical Approaches. J. Minim. Invasive Gynecol..

[51] Kim H., Shim S., Hwang Y., Kim M., Hwang H., Chung Y., Cho H.-H., Kim M.-R. (2018). Is robot-assisted laparoscopic myomectomy limited in multiple myomas? A feasibility for ten or more myomas. Obstet. Gynecol. Sci..

[52] Lee C.-Y., Chen I.H., Torng P.-L. (2018). Robotic myomectomy for large uterine myomas. Taiwan. J. Obstet. Gynecol..

[53] Takmaz O., Ozbasli E., Gundogan S., Bastu E., Batukan C., Dede S., Gungor M. (2018). Symptoms and Health Quality after Laparoscopic and Robotic Myomectomy. JSLS.

[54] Aendekerk S., Verguts J., Housmans S., Timmerman D. (2019). Implementing robotic assisted myomectomy in surgical practice—A retrospective cohort study. Gynecol. Surg..

[55] Choi S.H., Hong S., Kim M., Bae H.S., Kim M.K., Jung Y.W., Yun B.S., Seong S.J. (2019). Robotic-assisted laparoscopic myomectomy: The feasibility in single-site system. Obstet. Gynecol. Sci..

[56] Huberlant S., Lenot J., Neron M., Ranisavljevic N., Letouzey V., De Tayrac R., Masia F., Warembourg S. (2020). Fertility and obstetrical outcomes after robot-assisted laparoscopic myomectomy. Int. J. Med. Robot. Comput. Assist. Surg..

[57] Moawad G.N., Tyan P., Paek J., Tappy E.E., Park D., Choussein S., Srouji S.S., Gargiulo A. (2019). Comparison between single-site and multiport robot-assisted myomectomy. J. Robot. Surg..

[58] Movilla P., Orlando M., Wang J., Opoku-Anane J. (2020). Predictors of Prolonged Operative Time for Robotic-Assisted Laparoscopic Myomectomy: Development of a Preoperative Calculator for Total Operative Time. J. Minim. Invasive Gynecol..

[59] Sheu B.-C., Huang K.-J., Huang S.-C., Chang W.-C. (2020). Comparison of uterine scarring between robot-assisted laparoscopic myomectomy and conventional laparoscopic myomectomy. J. Obstet. Gynaecol..

[60] Lee S.R., Lee E.S., Lee Y.-J., Lee S.-W., Park J.Y., Kim D.-Y., Kim S.H., Kim Y.-M., Suh D.-S., Kim Y.-T. (2020). Robot-Assisted Laparoscopic Myomectomy versus Abdominal Myomectomy for Large Myomas Sized over 10 cm or Weighing 250 g. Yonsei Med. J..

[61] Park S.Y., Kim J., Jeong K., Jung S.I., Hur Y.M., Cho E.H., Moon H.-S., Chung H.W. (2020). Clinical experience of robotic myomectomy for fertility preservation using preoperative magnetic resonance imaging predictor. Obstet. Gynecol. Sci..

[62] Won S., Lee N., Kim M., Kim M.K., Jung Y.W., Yun B.S., Seong S.J. (2020). Comparison of operative time between robotic and laparoscopic myomectomy for removal of numerous myomas. Int. J. Med. Robot. Comput. Assist. Surg..

[63] Ahn S.H., Park J.H., Kim H.R., Cho S., Lee M., Seo S.K., Choi Y.S., Lee B.S. (2021). Robotic single-site versus multi-port myomectomy: A case–control study. BMC Surg..

[64] Özbaşlı E., Güngör M. (2021). Comparison of perioperative outcomes among robot-assisted, conventional laparoscopic, and abdominal/open myomectomies. J. Turk. Gynecol. Assoc..

[65] Park K.-M., Kang S., Kim C., Sung Y., Chung Y.-J., Song J., Kim S., Kim M.-R. (2021). Variables that prolong total operative time for robotic-assisted laparoscopic myomectomy: A 10-year tertiary hospital study in Korea. Eur. J. Obstet. Gynecol. Reprod. Biol..

[66] Goldberg H.R., McCaffrey C., Amjad H., Kives S. (2022). Fertility and Pregnancy Outcomes after Robotic-assisted Laparoscopic Myomectomy in a Canadian Cohort. J. Minim. Invasive Gynecol..

[67] Kim J.M., Lee Y.H., Chong G.O., Lee S.R., Hong D.G. (2022). Comparison of Multi- and Single-Site Robotic Myomectomy Using the Da Vinci^®^ SP Surgical System: A Propensity Score Matching Analysis. J. Clin. Med..

[68] Morales H.G., López R.R., López G.P., Mondragon P.J.C., Cortes D.V., Hernandez H.S., Guiot M.L., Camacho F.M.R. (2022). Surgical approach to uterine myomatosis in patients with infertility: Open, laparoscopic, and robotic surgery; results according to the quantity of fibroids. JBRA Assist. Reprod..

[69] Won S., Choi S.H., Lee N., Shim S.H., Kim M.K., Kim M.-L., Jung Y.W., Yun B.S., Seong S.J. (2022). Robotic Single-Site Plus Two-Port Myomectomy versus Conventional Robotic Multi-Port Myomectomy: A Propensity Score Matching Analysis. J. Pers. Med..

[70] Wang T., Tang H., Xie Z., Deng S. (2018). Robotic-assisted vs. laparoscopic and abdominal myomectomy for treatment of uterine fibroids: A meta-analysis. Minim. Invasive Ther. Allied Technol..

[71] Horn D., Sacarny A., Zhou A. (2022). Technology adoption and market allocation: The case of robotic surgery. J. Health Econ..

[72] Lim C.S., Mowers E.L., Mahnert N., Skinner B.D., Kamdar N., Morgan D.M., As-Sanie S. (2016). Risk Factors and Outcomes for Conversion to Laparotomy of Laparoscopic Hysterectomy in Benign Gynecology. Obstet. Gynecol..

[73] Lanfranco A.R., Castellanos A.E., Desai J.P., Meyers W.C. (2004). Robotic Surgery: A current perspective. Ann. Surg..

[74] Mucksavage P., Kerbl D.C., Lee J.Y. (2011). The da Vinci^®^ Surgical System Overcomes Innate Hand Dominance. J. Endourol..

[75] Heemskerk J., Zandbergen H.R., Keet S.W., Martijnse I., van Montfort G., Peters R.J., Svircevic V., Bouwman R.A., Baeten C.G., Bouvy N.D. (2014). Relax, It’s Just Laparoscopy! A Prospective Randomized Trial on Heart Rate Variability of the Surgeon in Robot-Assisted versus Conventional Laparoscopic Cholecystectomy. Dig. Surg..

[76] Hubert N., Gilles M., Desbrosses K., Meyer J.P., Felblinger J., Hubert J. (2013). Ergonomic assessment of the surgeon’s physical workload during standard and robotic assisted laparoscopic procedures. Int. J. Med. Robot. Comput. Assist. Surg..

[77] Arian S.E., Munoz J.L., Kim S., Falcone T. (2017). Robot-assisted laparoscopic myomectomy: Current status. Robot. Surg. Res. Rev..

