# Supplementary material for: Multi-Port Robotic-Assisted Laparoscopic Myomectomy: A Systematic Review and Meta-Analysis of Comparative Clinical and Fertility Outcomes
Source: J Clin Med. 2023 Jun 19;12(12):4134. doi: 10.3390/jcm12124134 (PMC10299339; doi:10.3390/jcm12124134)
Supplement: Supplementary file 1 [file jcm-12-04134-s001.zip › jcm-2309212-supplementary.pdf]

Supplementary Table S1: Baseline characteristics of the included studies (N/A: not applicable, RALM: robotic as-sisted laparoscopic myomectomy, CLM: conventional laparoscopic myomectomy, AM: abdominal myomectomy).

Study	Publication Year	Recruitment period	Country	Study design	Participants	Patient groups	Comparator	Clinical data	Fertility data
Advincula et al.[17]	2007	2000- 2004	USA	retrospective	58	2	AM	Yes	No
Bedient et al.[18]	2009	2000-2008	USA	retrospective	81	2	CLM	Yes	No
George et al.[19]	2009	2005-2008	USA	retrospective	77	1	N/A	Yes	No
Nezhat et al.[20]	2009	2006 -2007	USA	retrospective	50	2	CLM	Yes	No
Piquion-Joseph et al.[21]	2009	2005-2008	USA	retrospective	18	1	N/A	Yes	No
Ascher-Walsh et al.[22]	2010	2005-2008	USA	retrospective	125	2	AM	Yes	No
Sangha et al.[23]	2010	2005-2009	USA	retrospective	148	2	AM	Yes	No
Barakat et al.[24]	2011	1995-2009	USA	retrospective	575	3	CLM, AM	Yes	No
Nash et al.[25]	2012	2008-2010	USA	retrospective	81	2	AM	Yes	No
Lonnerfors et al.[26]	2011	2006-2010	Sweden	prospective	31	1	N/A	Yes	Yes
Cela et al.[27]	2012	2007-2011	Italy	prospective	48	1	N/A	Yes	Yes
Gargiulo et al.[28]	2012	2007-2009	USA	retrospective	289	2	CLM	Yes	No
Gocmen et al.[29]	2012	2008-2010	Turkey	retrospective	38	2	CLM	Yes	No
Hsiao et al.[30]	2012	2010-2011	Taiwan	retrospective	42	2	CLM	Yes	No
Mansour et al.[31]	2012	2008-2011	Canada	retrospective	59	2	AM	Yes	No
Pitter et al.[32]	2012	2005-2010	USA	retrospective	107	1	N/A	Yes	No
Tan et al.[33]	2012	2009-2011	Taiwan	retrospective	13	1	N/A	Yes	No
Tusheva et al.[34]	2012	2006-2009	USA	retrospective	30	1	N/A	Yes	Yes
Gobern et al.[35]	2013	2007-2009	USA	retrospective	308	3	CLM, AM	Yes	No

Griffin et al.[36]	2013	2011-2012	USA	prospective	39	2	AM	Yes	No
Pluchino et al.[37]	2013	2009-2012	Italy	retrospective	86	2	CLM	Yes	No
Goetgheluck et al.[38]	2014	2010-2012.	France	retrospective	18	1	N/A	Yes	No
Hanafi et al.[39]	2014	2007-2009	USA	prospective	122	2	AM	Yes	No
Asmar et al.[40]	2015	2011-2014	France	retrospective	36	1	N/A	Yes	Yes
Cheng et al.[41]	2015	2010-2012	Taiwan	prospective	22	1	N/A	Yes	No
Yim et al.[42]	2015	2006-2013	Korea	retrospective	17	1	N/A	Yes	No
Pitter et al.[43]	2015	2005-2013	USA	retrospective	426	1	N/A	No	Yes
Gunnala et al.[44]	2016	2010-2013	USA	retrospective	207	1	N/A	Yes	No
Kang et al.[45]	2016	2009-2013	Korea	retrospective	100	1	N/A	Yes	Yes
Flyckt et al.[46]	2016	1995-2009	USA	retrospective	15	1	N/A	No	Yes
Chen et al.[47]	2017	2011-2015	Taiwan	retrospective	122	1	N/A	Yes	No
Nam et al.[48]	2017	2016	Korea	prospective	15	1	N/A	Yes	No
Chen et al.[49]	2018	2012-2016	Taiwan	retrospective	78	2	CLM	Yes	No
Jansen et al.[50]	2018	2009-2016	USA	retrospective	659	3	CLM, AM	Yes	No
Kim et al.[51]	2018	2010-2016.	Korea	retrospective	26	2	AM	Yes	No
Lee et al.[52]	2018	2012-2017	Taiwan	retrospective	74	1	N/A	Yes	No
Takmaz et al.[53]	2018	2016-2017	Turkey	prospective	64	2	CLM	Yes	No
Aendekerk et al.[54]	2019	2009-2016	Belgium	retrospective	187	3	CLM, AM	Yes	Yes
Choi et al.[55]	2019	2015-2018	Korea	retrospective	250	1	N/A	Yes	No
Huberland et al.[56]	2019	2009-2016	France	retrospective	53	1	N/A	Yes	Yes
Moawad et al.[57]	2019	2011-2016	USA	retrospective	95	1	N/A	Yes	No
Movilla et al.[58]	2019	2014-2017	USA	retrospective	126	1	N/A	Yes	No
Sheu et al.[59]	2019	2014-2017	Taiwan	prospective	203	2	CLM	Yes	Yes
Lee SR et al.[60]	2020	2019-2020	Korea	retrospective	277	2	AM	Yes	No

Park SY et al.[61]	2020	2015-2018	Korea	retrospective	55	1	N/A	Yes	Yes
Won et al.[62]	2020	2017-2019	Korea	retrospective	265	2	CLM	Yes	No
Ahn et al.[63]	2021	2016-2021	Korea	retrospective	90	1	N/A	Yes	No
Ozbasli et al.[64]	2021	2016-2020	Turkey	retrospective	227	3	CLM, AM	Yes	No
Park KM et al.[65]	2021	2009-2019	Korea	retrospective	584	1	N/A	Yes	No
Goldberg et al.[66]	2021	2008-2015	Canada	retrospective	101	1	N/A	No	Yes
Kim et al.[67]	2022	NR	Korea	retrospective	50	1	N/A	No	Yes
Morales et al.[68]	2022	2010-2018	Mexico	retrospective	69	3	CLM, AM	Yes	Yes
Won et al.[69]	2022	2020-2021	Korea	retrospective	173	1	N/A	Yes	No

Supplementary Table S2: Baseline and clinical patient characteristics. (NR: not reported, *the same study divided in 2 because of different groups, RALM: robotic assisted laparoscopic myomectomy, CLM: conventional laparoscopic myomectomy, AM: abdominal myomectomy).

Study	Year	Patients	Method	Mean age (±SD)	BMI (±SD)	Parity		CS and/or AS history	Clinical manifestations						Co-morbidities
						0	1+		Mass	Pain	Bleeding	Infertility	Other	None	
Advincula et al.[17]	2007	29	RALM	36.6±4.9	25.2±3.9	NR	NR	NR	12	5	15	2	20	0	NR
		29	AM	34.9±4.4	28.3±6.7	NR	NR	NR	7	1	15	9	16	0	NR
Bedient et al.[18]	2009	40	RALM	43±12	24.7±5.0	NR	NR	NR	NR	NR	NR	NR	NR	NR	NR
		41	CLM	40.9±6.6	25.3±5.4	NR	NR	NR	NR	NR	NR	NR	NR	NR	NR
George et al.[19]	2009	77	RALM	37.1±5	29.1±6.3	NR	NR	NR	NR	NR	NR	NR	NR	NR	NR
Nezhat et al.[20]	2009	15	RALM	39±6.3	23.0±3.7	NR	NR	5	NR	NR	NR	NR	NR	NR	NR
		35	CLM	41±6.4	24.0±3.3	NR	NR	15	NR	NR	NR	NR	NR	NR	NR
Piquion-Joseph et al.[21]	2009	18	RALM	36±7	29.0±6.0	NR	NR	4	NR	NR	NR	NR	NR	NR	6
Ascher-Walsh et al.[22]	2010	75	RALM	36.5±7.2	21.7±3.7	NR	NR	NR	NR	NR	NR	NR	NR	NR	NR
		50	AM	37.2±5.4	20.4±4.1	NR	NR	NR	NR	NR	NR	NR	NR	NR	NR
Sangha et al.[23]	2010	100	RALM	36.2±5.5	29.1	NR	NR	NR	NR	NR	NR	NR	NR	NR	NR
		48	AM	36.4±6.8	29.1	NR	NR	NR	NR	NR	NR	NR	NR	NR	NR
Barakat et al.[24]	2011	89	RALM	37±5.7	25.8±5.3	59	28	28	NR	NR	NR	NR	NR	NR	NR
		93	CLM	38.2±1.8	25.1±6.0	51	41	59	NR	NR	NR	NR	NR	NR	NR
		393	AM	37±1.4	28.2±7.5	243	187	132	NR	NR	NR	NR	NR	NR	NR
Nash et al.[25]	2012	27	RALM	38.3±6.3	25.0±4.8	NR	NR	NR	0	21	14	0	8	0	NR
		54	AM	35.8±5.5	26.5±6.2	NR	NR	NR	0	31	40	0	7	0	NR
Lonnerfors et al.[26]	2011	31	RALM	35± 3.4	22.1±1.1	21	10	NR	NR	NR	NR	14	NR	NR	NR
Cela et al.[27]	2012	48	RALM	35.2± 6.0	23.5±4.1	31	17	NR	NR	NR	NR	NR	NR	NR	NR
Gargiulo et al.[28]	2012	174	RALM	37.8±6.5	25.9±6.4	NR	NR	10	NR	NR	NR	NR	NR	NR	NR
		115	CLM	39.2±5.7	26.2±7.3	NR	NR	10	NR	NR	NR	NR	NR	NR	NR
Gocmen et al.[29]	2012	15	RALM	34.2±5.7	25.6 ±3.3	NR	NR	NR	NR	NR	NR	NR	NR	NR	NR
		23	CLM	35.7±6.1	27.6±5.2	NR	NR	NR	NR	NR	NR	NR	NR	NR	NR
	2012	20	RALM	45.8±4.8	24.2±2.2	NR	NR	6	1	2	13	0	9	NR	NR

Hsiao et al.[30]		22	CLM	46.6±3.2	23.4±2.5	NR	NR	12	2	3	13	0	14	NR	NR
Mansour et al.[31]	2012	38	RALM	34.7±4.4	25.1±4.8	30	8	8	18		17	2	1	0	NR
		21	AM	35.3±4.7	26.5±5.5	19	2	7	10		6	1	1	1	NR
Pitter et al.[32]	2012	107	RALM	34.8±4.5	27.4±5.5	92	12	40	NR	NR	NR	NR	NR	NR	NR
Tan et al.[33]	2012	13	RALM	38.3 ±6.3	20.2 ±1.9	NR	NR	5	NR	NR	NR	NR	NR	NR	6
Tusheva et al.[34]	2012	30	RALM	34.1±5.4	NR	NR	NR	NR	NR	NR	NR	NR	NR	NR	NR
Gobern et al.[35]	2013	66	RALM	39.8±5.3	28.3±7.2	48	18	24	23		23	23	7	NR	6
		73	CLM	39.1±6.9	26.5±6.9	57	16	30	35		24	19	10	NR	37
		169	AM	39.0±4.3	27.4±5.2	143	26	70	55		66	92	5	NR	13
Griffin et al.[36]	2013	16	RALM	33.8±5.0	23.6±3.8	NR	NR	NR	0	7	7	10		0	NR
		23	AM	35.2±5.0	27.3±7.5	NR	NR	NR	0	5	17	6		0	NR
Pluchino et al.[37]	2013	43	RALM	34.7±6.0	23.0±3.7	NR	NR	9	NR	NR	NR	NR	NR	NR	NR
		43	CLM	36.4±7.1	23.8±2.4	NR	NR	12	NR	NR	NR	NR	NR	NR	NR
Goetgheluck et al.[38]	2014	18	RALM	37.9±5.2	28±4.1	3	15	1	NR	NR	NR	NR	NR	NR	NR
Hanafi et al.[39]	2014	77	RALM	38.4±6.6	28.1±6.0	NR	NR	NR	NR	NR	NR	NR	NR	NR	NR
		45	AM	37.0±5.6	31.0±7.2	NR	NR	NR	NR	NR	NR	NR	NR	NR	NR
Asmar et al.[40]	2015	36	RALM	39.1±8.0	NR	NR	NR	10	1	24		6	NR	NR	
Cheng et al.[41]	2015	22	RALM	40.1±4.5	24.1±4.4	NR	NR	4	NR	NR	NR	NR	NR	NR	3
Yim et al.[42]	2015	17	RALM	48.2±9.4	23.3±4.0	NR	NR	NR	NR	NR	NR	NR	NR	NR	NR
Pitter et al.[43]	2015	426	RALM	37.9±5.8	27.0±6.0	NR	NR	62	177		192	68	79	0	191
Gunnala et al.(i)[44]*	2016	141	RALM	35.6±5.8	26.3±6.1	NR	NR	75	NR	NR	NR	NR	NR	NR	NR
Gunnala et al.(ii)[44]	2016	66	RALM	36.9±5.4	24.7±6.0	NR	NR	17	NR	NR	NR	NR	NR	NR	NR
Kang et al.[45]	2016	100	RALM	35.3±5.6	21.8±3.0	86	14	NR	NR	NR	NR	NR	NR	NR	32
Flyckt et al.[46]	2016	15	RALM	34.0±3.8	29.7±6.2	NR	NR	NR	NR	NR	NR	NR	NR	NR	NR
Chen et al.[47]	2017	122	RALM	46.9±13.1	23.0±5.5	NR	NR	NR	NR	NR	NR	NR	NR	NR	NR
Nam et al.[48]	2017	15	RALM	39.1±5.5	22.1±1.9	7	8	5	0	1	3	0	11	NR	NR
Chen et al.[49]	2018	26	RALM	42.1±5.5	23.6±4.6	NR	NR	3	7	7	14	0	24	0	NR
		52	CLM	46.7±3.8	25.3±5.0	NR	NR	18	7	7	34	0	36	0	NR

Jansen et al.[50]	2018	163	RALM	38.4±6.8	27.2±5.8	NR	NR	33	97		71	.42	64	0	NR
		185	CLM	39.1±7.3	27.2±5.7	NR	NR	37	148		106	15	73	0	NR
		311	AM	38.1±5.9	27.8±6	NR	NR	77	187		182	88	109	0	NR
Kim et al.[51]	2018	13	RALM	37.0±3.3	21.6±2.5	NR	NR	3	NR	NR	NR	NR	NR	NR	NR
		13	AM	38.1±3.6	23.4±3.0	NR	NR	1	NR	NR	NR	NR	NR	NR	NR
Lee et al.(i)*[52]	2018	42	RALM	39.9±7.3	21.9±2.9	32	10	13	1		19	0	7	8	17
Lee et al.(ii)[52]	2018	32	RALM	37.1±5.2	21.7±2.4	29	3	5	4		13	0	13	5	6
Takmaz et al.[53]	2018	31	RALM	38.0±5.0	23.0±4.0	NR	NR	NR	NR	NR	NR	NR	NR	NR	NR
		33	CLM	35.0±5.0	24.0±4.0	NR	NR	NR	NR	NR	NR	NR	NR	NR	NR
Aendekerk et al.[54]	2019	51	RLM	34.0±8.2	25.0±5.1	NR	NR	NR	NR	NR	NR	NR	NR	NR	NR
		84	CLM	37.0±7.0	24.0±4.1	NR	NR	NR	NR	NR	NR	NR	NR	NR	NR
		52	AM	35.0±8.4	25.0±4.2	NR	NR	NR	NR	NR	NR	NR	NR	NR	NR
Choi et al.[55]	2019	250	RALM	37.6±5.4	22.5±3.2	199	51	35	NR	NR	NR	NR	NR	NR	62
Huberland et al.[56]	2019	53	RALM	35.4±5.3	25.5±4.3	NR	NR	22	0	29	16	34	31	0	NR
Moawad et al.[57]	2019	95	RALM	36.1±5.7	27.9±6.3	NR	NR	16	NR	NR	NR	NR	NR	NR	NR
Movilla et al.[58]	2019	126	RALM	39.4±7.0	25.7±5.8	91	35	42	45	5	66	4	0	0	13
Sheu et al.[59]	2019	93	RALM	39 ± 6.7	21.9±2.9	NR	NR	NR	NR	NR	NR	NR	NR	NR	NR
		110	CLM	39 ± 6.1	22.4±3.5	NR	NR	NR	NR	NR	NR	NR	NR	NR	NR
Lee SR et al.[60]	2020	126	RALM	38.1±5.9	23.1±4.1	NR	NR	23	NR	NR	NR	NR	NR	NR	6
		151	AM	38.1±5.6	23.5±4.2	NR	NR	41	NR	NR	NR	NR	NR	NR	8
Park SY et al.[61]	2020	55	RALM	35.7±5.0	NR	NR	NR	NR	NR	NR	NR	NR	NR	NR	NR
Won et al.[62]	2020	121	RALM	39.1±5.8	22.7±3.0	95	26	29	NR	NR	NR	NR	NR	NR	31
		144	CLM	39.3 ± 5.6	22.9 ±4.1	103	41	46	NR	NR	NR	NR	NR	NR	40
Ahn et al.[63]	2021	90	RALM	37.5 ± 5.7	NR	NR	NR	NR	NR	NR	NR	NR	NR	NR	NR
Ozbasli et al.[64]	2021	66	RALM	38.6±5.8	22.6±2.5	41	25	46	15	11	34	2	4	0	NR
		88	CLM	38.0±5.4	23.4±3.9	53	35	72	17	30	31	0	10	0	NR
		73	AM	38.9±5.3	24.7±4.5	55	18	62	1	14	35	18	5	0	NR
Park KM et al.[65]	2021	584	RALM	36.3±5.5	22.2±3.2	NR	NR	100	50	62	162	19	291	0	9

Goldberg et al.[66]	2021	101	RALM	34.4±4.4	25.2±1.3	NR	NR	NR	30		40	30	0	1	NR
Kim et al.[67]	2022	50	RALM	36.3±7.2	21.4±2.3	30	NR	6	NR	NR	NR	NR	NR	NR	NR
Morales et al.[68]	2022	24	RALM	35.2±4.2	23.4±1.8	NR	NR	6	NR	NR	NR	NR	NR	NR	NR
		24	CLM	37.2±5.7	24.6±3.3	NR	NR	3	NR	NR	NR	NR	NR	NR	NR
		21	AM	36.9±4.5	25.6±4.0	NR	NR	6	NR	NR	NR	NR	NR	NR	NR
Won et al.[69]	2022	173	RALM	38.4±5.4	23.2±3.3	148	25	24	NR	NR	NR	NR	NR	NR	25

Supplementary Table S3: Da Vinci surgical system and procedure characteristics, fibroid baseline characteristics (MMN: Mean Myoma Number, MMD: Mean Myoma Diameter, MDLM: Mean Diameter of Largest Myomas, MMW: Mean Myoma Weight, NR: not reported).

Study	Year	Patients	Da Vinci System	Camera port (mm)	Robotic ports (mm)	Accessory port(s) (mm)	Mean Myoma Number (MMN)	Mean Myoma Diameter (MMD)	Mean Diameter of Largest Myomas (MDLM)	Mean Myoma Weight (MMW)
Advincula et al.[17]	2007	29	Si	12	8,8	12	NR	NR	NR	227.86±247.54
Bedient et al.[18]	2009	41	Standard or S	NR	NR	NR	2.7±1.9	NR	NR	210+-270
George et al.[19]	2009	77	Standard	NR	NR	NR	NR	NR	NR	270.42±199.35
Nezhat et al.[20]	2009	15	Standard	12	8,8	5 or 12	3.0±1.7	NR	5.1±1.30	116±93.42
Piquion-Joseph et al.[21]	2009	18	Standard	12	8,8,(8)	12	NR	NR	NR	NR
Ascher-Walsh et al.[22]	2010	125	Si	12	8,8	12	2.4±1.5	NR	NR	321.16±243.87
Sangha et al.[23]	2010	100	Standard	12	8,8	5 or 10	3.0±3.8	NR	7.85±3.45	NR
Barakat et al.[24]	2011	89	Si	12	8,8,8	10	2.9±1.7	7.9±3.0	NR	270.3±245.2
Nash et al.[25]	2012	27	Standard	NR	NR	NR	NR	NR	NR	NR
Lonnerfors et al.[26]	2011	31	Si	12	8,8,8	12 or 15	1.5±1.0	7.12±1.7	NR	NR
Cela et al.[27]	2012	48	Si	12	8,8	12	1.5±1.4	6.7±1.9	NR	NR
Gargiulo et al.[28]	2012	174	Standard	12	8,8,(8)	12	3.4±2.8	NR	NR	177.11±143.6
Gocmen et al.[29]	2012	15	S	12	8,8	12	2.73 ± 3.10	NR	6.00 ± 1.50	NR
Hsiao et al.[30]	2012	20	Si	12	8,8	10	2.36±2.4	NR	6.41±1.52	139.3±145.2
Mansour et al.[31]	2012	38	Standard	12	8,8	12 or 15	2.0 ± 1.4	NR	9.1 ± 2.0	389.0 ± 170.4
Pitter et al.[32]	2012	108	Standard	NR	NR	NR	3.9±3.2	NR	7.5±3.0	191.7±144.8
Tan et al.[33]	2012	13	Standard	NR	NR	NR	NR	NR	NR	119.6 66.2
Tusheva et al.[34]	2012	30	S	12	7,7	8 and/or 5	2.24±0.98	NR	7.21±3.7	NR
Gobern et al.[35]	2013	66	Standard	NR	NR	NR	NR	NR	6.9±3.54	179.6±197.1
Griffin et al.[36]	2013	16	Standard	NR	NR	NR	2.8±1.9	NR	NR	318.6±154
Pluchino et al.[37]	2013	43	Si	12	8,8	10	1.97±1.48	4.76±1.71	NR	117.8±30.05
Goetgheluck et al.[38]	2014	18	Si	12	8,8	12	1.34±0.58	7.7±2.74	NR	149.7±77.7
Hanafi et al.[39]	2014	77	S	12	8,8	12	3.1±1.4	4.3±1.79	NR	NR
Asmar et al.[40]	2015	36	Si	NR	NR	NR	1.56±1.04	7.5±3.3	NR	172.3±92
Cheng et al.[41]	2015	22	Si	12	8,8,8	12	3.1±4.1893	NR	NR	367.4 ± 317.7

Yim et al.[42]	2015	17	S or Si	12	8,8	5 or 10	NR	NR	NR	NR
Pitter et al.[43]	2015	426	Multiple models	NR	NR	NR	NR	NR	NR	NR
Gunnala et al. (i)[44]	2016	141	S	12	8,8,8	5	NR	NR	NR	106 ± 164.5
Gunnala et al. (ii)[44]	2016	66	S	12	8,8,8	5	NR	NR	NR	510 ± 379.5
Kang et al.[45]	2016	100	S	12	8,8	5	3.8±3.5	NR	7.5±2.1	NR
Flyckt et al.[46]	2016		Standard	12	8,8	12 or 5	NR	NR	NR	NR
Chen et al.[47]	2017	122	Si	12	8,8	NR	NR	NR	NR	NR
Nam et al.[48]	2017	15	Xi	12	8,8,(8)	5	3.2±2.4	NR	7.60±1.67	180.5±58.7
Chen et al.[49]	2018	26	Si	12	8,8,(8)	10	2.8222±2.902	NR	NR	217.15±125.4933
Jansen et al.[50]	2018	163	Standard	NR	NR	NR	6.7±4.7	NR	9±3.4	399.6±250.1
Kim et al.[51]	2018	13	S	12	8,8	5	13.7±3.0	34.7±10.31	6.8±1.4944	229.07±98.9577
Lee et al. (i)[52]	2018	42	Si or Xi	12	8,8,(8)	12	3.6 ± 3.3	NR	7.2 ± 1.7	288.1 ± 147.5
Lee et al. (ii)[52]	2018	32	Si or Xi	12	8,8,(8)	12	3.6 ± 3.1	NR	11.3 ± 2.3	446.5 ± 206.2
Takmaz et al.[53]	2018	31	Xi	NR	NR	NR	2.48±1.011	NR	6.8±1.1	NR
Aendekerk et al.[54]	2019	51	SI	NR	NR	NR	2.26±1.33	7.0±2.24	NR	NR
Choi et al.[55]	2019	250	SI	12	8,8	5	4.6±4.1	NR	NR	250.8±208.1
Huberland et al.[56]	2019	53	Standard	NR	NR	NR	2±1.57	NR	6.9±1.77	173±129.4
Moawad et al.[57]	2019	95	Multiple models	12	8,8,(8)	(12)	4.7±4.11	8.3±3.85	NR	342.8±325.8
Movilla et al.[58]	2019	126	Xi	NR	NR	NR	2.7±2.4	NR	8.4±2.6	264.4±236.2
Sheu et al.[59]	2019	93	Si	12	8,8	12	3.3 ± 3.4	NR	9.0 ± 2.6	322 ± 208
Lee SR et al.[60]	2020	126	Si or Xi	NR	NR	NR	4.394±6.3972	NR	10.8±2.52	444.6±283.14
Park SY et al.[61]	2020	55	Si	NR	NR	NR	5.36±5.27	NR	NR	360.78±318.35
Won et al.[62]	2020	121	Si	12,8,8,10	8,8	10	5.7 ± 5.1	NR	7.7 ± 4.2	234.3 ± 174.0
Ahn et al.[63]	2021	236	SI	12,8,8,10	8,8	10	4.1 ± 3.7	7.7 ± 3	NR	NR
Ozbasli et al.[64]	2021	66	Xi	8	8,8	12	3.4±2.13	NR	8.44±2.98	237.2±309.61
Park KM et al.[65]	2021	584	Xi or S	NR	NR	NR	NR	NR	NR	202.2±152.6
Goldberg et al.[66]	2021	101	Standard	NR	NR	NR	NR	NR	NR	NR

Kim et al.[67]	2022	50	Si or Xi	12	8,8	NR	3.90±3.72	NR	NR	251.66±213.57
Morales et al.[68]	2022	24	Si	NR	NR	NR	3.85±3.06	NR	5.61±4.63	33.84±76.70
Won et al.[69]	2022	173	Si or xi	12	8,8	12	7.1±5.3	NR	7.0±2.5	204.0±147.2
